# Supplementary material for: Looking for pathways related to COVID-19: confirmation of pathogenic mechanisms by SARS-CoV-2–host interactome
Source: Cell Death Dis. 2021 Aug 12;12(8):788. doi: 10.1038/s41419-021-03881-8 (PMC8357963; doi:10.1038/s41419-021-03881-8)
Supplement: Supplementary file 1 — Supplementary Figure Legends [file 41419_2021_3881_MOESM1_ESM.docx]

**Supplementary Figure Legends**

**Supplementary Table 1.** Human and viral proteins identified by RWR in all SARS-CoV-2 proteins interactome and represented in Fig.1, along with statistical parameters and biological information.

**Supplementary Table 2**. Gene enrichment analysis obtained by KEGG 2019 database. Results were considered significant with a p-value < 0.05.

**Supplementary Table 3**. Gene enrichment analysis obtained by WikiPathways 2019 database. Results were considered significant with a p-value < 0. 05.

**Supplementary Table 4**. All Interactions selected by RWR in interactome for single seed viral protein. Detailed information about interactors, interaction and database were reported.

**Supplementary Figure 1.** KEGG human pathways and WikiPathways Gene Enrichment analyses for 200 proteins identified by RWR algorithm using together 28 proteins of SARS-CoV-2.

**Supplementary Figure 2.** Plot betweenness centrality vs. degree of 199 human proteins, retained in all proteins of SARS-CoV-2 – host interactome. Limits were fixed to top 5% of Betweenness Centrality values and Degree, 0. 020554 and 11 respectively.

**Supplementary Figure 3.** Interactomes based on human PPI and SARS-CoV-2 – host interactions, with top 50 closest proteins identified by RWR, using structural proteins (M, S, N, E) as seed. The nodes in red are human proteins, while the nodes in green are virus proteins.

**Supplementary Figure 4.** Interactomes based on human PPI and SARS-CoV-2 – host interactions, with top 50 closest proteins identified by RWR, using accessory proteins (ORF1a, ORF3a, ORF6, ORF7a, NS7b, ORF8, ORF9b, ORF10, ORF14) as seed. The nodes in red are human proteins, while the nodes in green are virus proteins.

**Supplementary Figure 5.** Interactomes based on human PPI and SARS-CoV-2 – host interactions, with top 50 closest proteins identified by RWR, using accessory proteins (nsp1, nsp2, nsp3, nsp4, nsp5, nsp6, nsp7, nsp8, nsp9, nsp10, nsp12, nsp13, nsp14, nsp15, nsp16) as seed. The nodes in red are human proteins, while the nodes in green are virus proteins.
