# Supplementary figures and images for: Looking for pathways related to COVID-19: confirmation of pathogenic mechanisms by SARS-CoV-2–host interactome
Source: Cell Death Dis. 2021 Aug 12;12(8):788. doi: 10.1038/s41419-021-03881-8 (PMC8357963; doi:10.1038/s41419-021-03881-8)

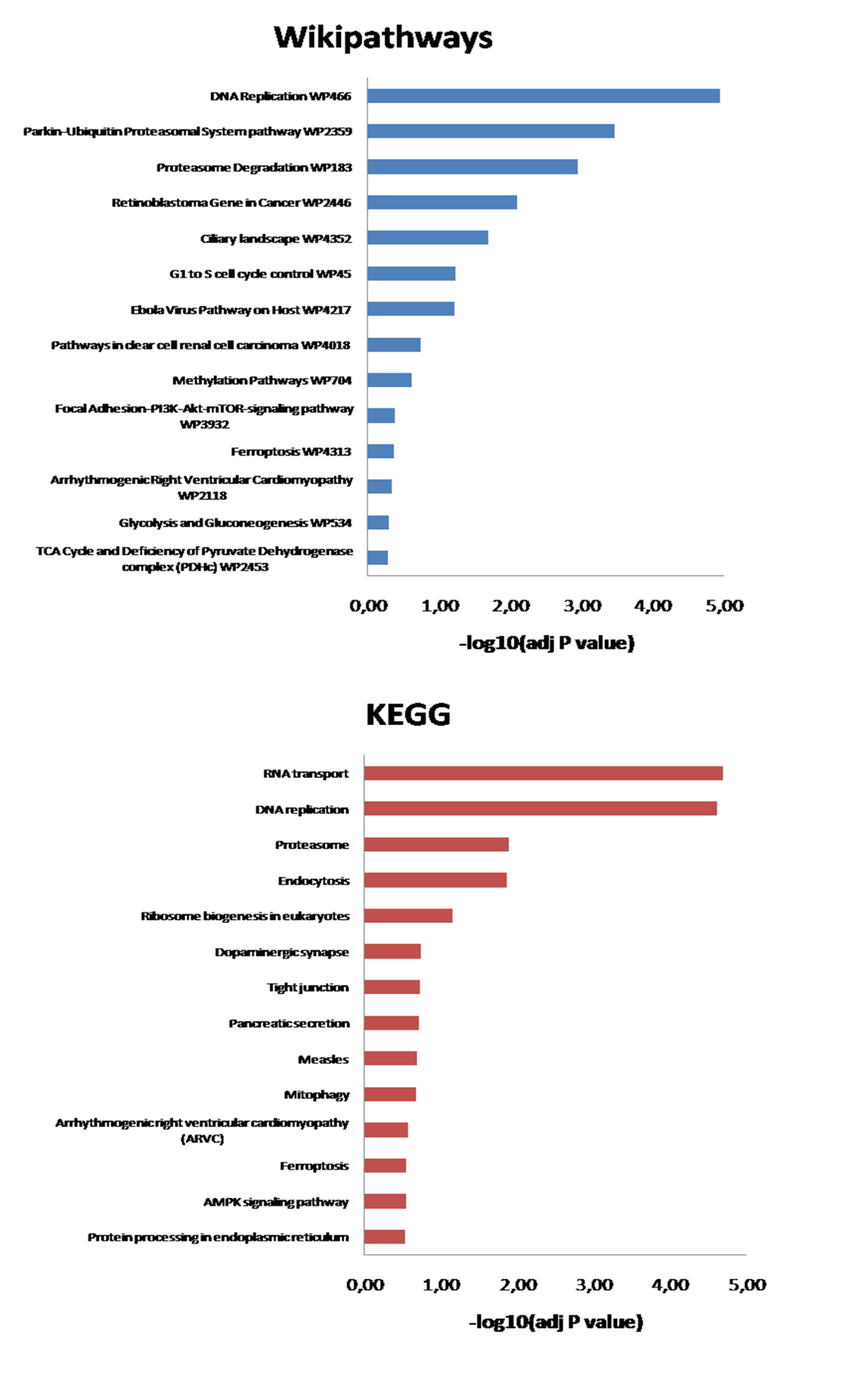

Supplement: Supplementary file 2 — Supplementary Figure 1 [file 41419_2021_3881_MOESM2_ESM.tif]

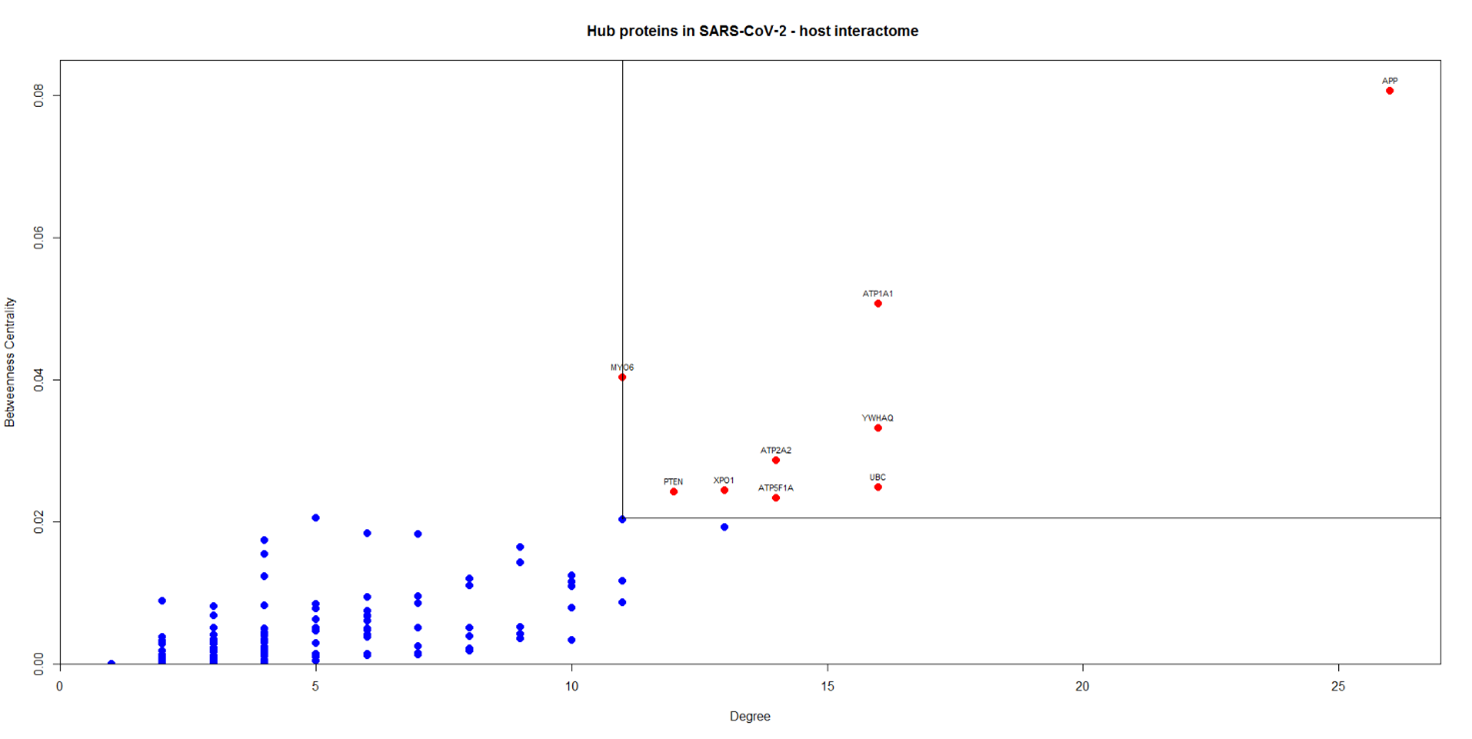

Supplement: Supplementary file 3 — Supplementary Figure 2 [file 41419_2021_3881_MOESM3_ESM.tif]

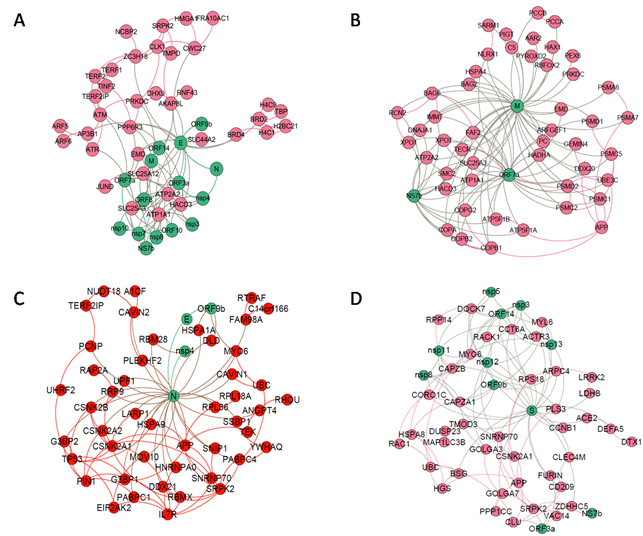

Supplement: Supplementary file 4 — Supplementary Figure 3 [file 41419_2021_3881_MOESM4_ESM.tif]

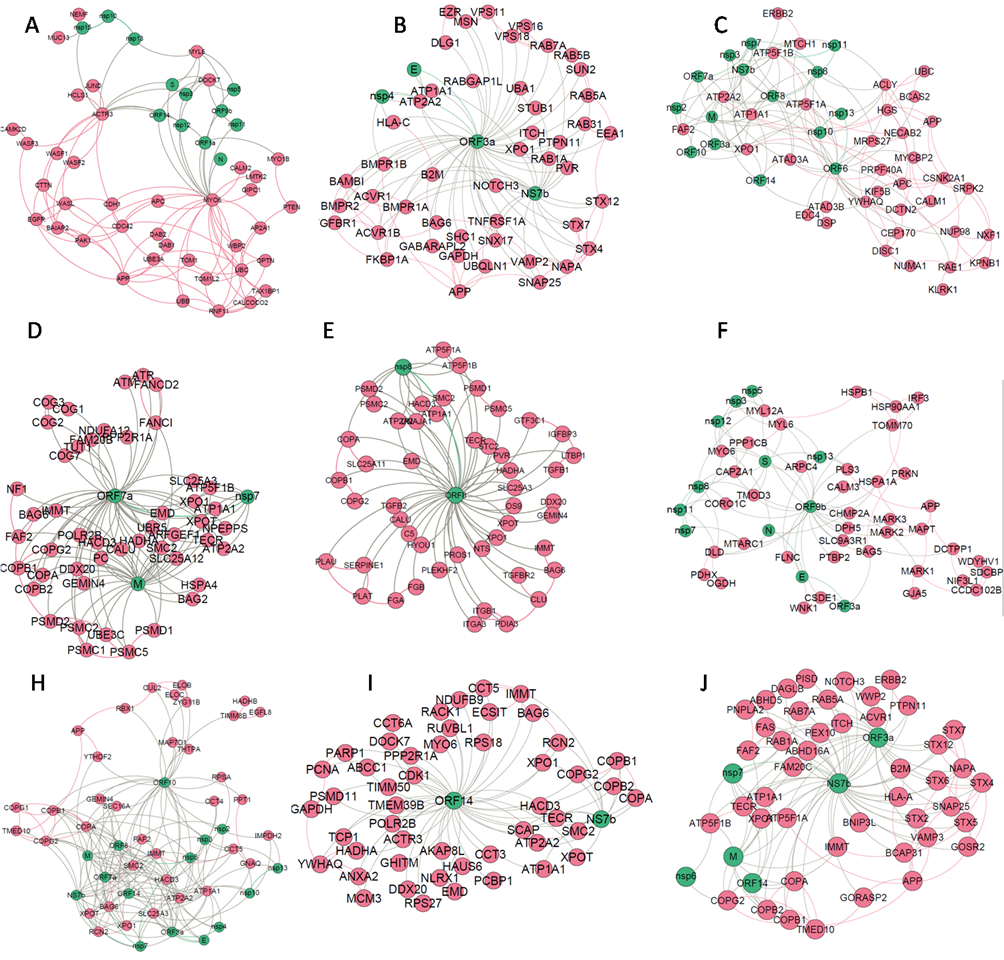

Supplement: Supplementary file 5 — Supplementary Figure 4 [file 41419_2021_3881_MOESM5_ESM.tif]

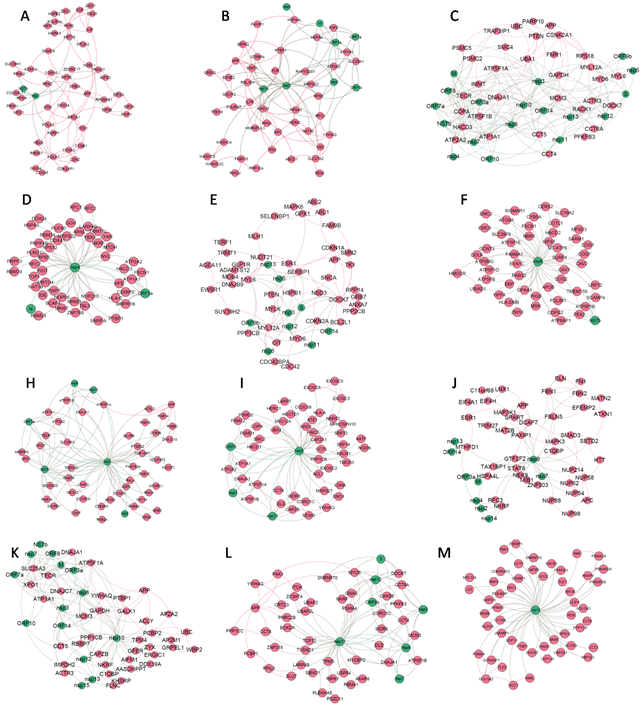

Supplement: Supplementary file 6 — Supplementary Figure 5 [file 41419_2021_3881_MOESM6_ESM.tif]
